# Malaria

**Published:** 1876-09

**Authors:** Charles P. Russel


Let us now consider under what circumstances malaria may be produced. Although it can not be denied that there are peculiar localities where, with apparently every presumed condition existing for the development of malaria, that poison is entirely absent, yet the concurrence of malarial emanations with such conditions in innumerable places establishes beyond a question their direct relation. The *essential* element in the production of malaria would appear to be *vegetable decomposition;* and, in order that this process shall ensue, the simultaneous operation of *air*, *moisture*, and *a certain high range of temperature*, is absolutely required. Localities, therefore, where such combination occurs, are prolific of malaria. Of this character are swamps and morasses, alluvial deposits, loose, porous, sandy, and argillaceous soils, or deep, loamy, marly lands underlaid by impermeable strata affording capacity for the retention of moisture, regions exposed to periodical or occasional inundation, places left bare by the subsidence of lakes or drying up of streams, and particularly areas subject to the intermingling of salt and fresh water—as salt marshes into which fresh streams discharge, or regions liable to tidal overflow and recession.

The exhalations from marshy tracts are recognized by their effects upon the human system throughout the world; and the fact that marshes bear a causative relation to malaria has been demonstrated in numerous instances by the disappearance of fever after thorough drainage and cultivation, and its reappearance upon their being allowed to relapse into neglect. The favorable effect of drainage and cultivation is owing both to the systematic removal of water near the surface, and most probably also to the absorption by the growing crops of the products of organic decomposition. On the same principle, Prof. Maury succeeded in anta onizing the noxious emanations from a marsh surrounding the observatory at Washington by planting it thickly with sun flowers, which seem to possess an extraordinary absorbing power. Sebastian is inclined to believe that the *Calamus aromaticus* which grows in some swamps has a similar neutralizing quality. Swamps covered with water are not so dangerous as those partially dry, the layer of water serving as a protection against the access of air and heat to the vegetable matter underneath.

A certain continuous range of temperature seems essential to the development of malaria, which is almost unknown beyond 60° north and 57° south latitude, and during the cold season in the Temperate Zone. According to Hirsch, it prevails up to various degrees of latitude and average annual temperature. It is the average summer temperature, however, that is of account, and the northern limit of this lies between the isotherms of 59° and 59.8° Fahr., giving a prolonged temperature sufficiently high to insure vegetable decomposition.—Dr. Charles. P. Russel, *in Popular Science Monthly for August.*

